# Development and validation of a machine-learning model for predicting the risk of death in sepsis patients with acute kidney injury

**DOI:** 10.1016/j.heliyon.2024.e29985

**Published:** 2024-04-20

**Authors:** Lei Dong, Pei Liu, Zhili Qi, Jin Lin, Meili Duan

**Affiliations:** Department of Critical Care Medicine, Beijing Friendship Hospital, Capital Medical University, Beijing 100050, China

**Keywords:** Machine learning, Sepsis, Acute kidney injury, Mortality

## Abstract

The mortality rate of patients with sepsis-induced acute kidney injury (S-AKI) is notably elevated. The initial categorization of prognostic indicators has a beneficial impact on elucidating and enhancing disease outcomes. This study aimed to predict the mortality risk of S-AKI patients by employing machine learning techniques. The sample size determined by a four-step procedure yielded 1508 samples. The research design necessitated the inclusion of individuals with S-AKI from the Medical Information Mart for Intensive Care (MIMIC)-IV database. The patients were initially admitted to the Intensive Care Unit (ICU) for their hospital stay. Additionally, these patients (aged from 18 to 89 years old) had encountered S-AKI on the day of their admittance. Forty-two predictive factors were analyzed, with hospitalization death as the outcome variable. The training set (4001 cases) consisted of 70 % of the participants, and the remaining (1714 cases) participants were allocated to the validation set. Furthermore, an additional validation set (MIMIC-III) consisted of 1757 patients from the MIMIC-III database. Moreover, an external validation set from the Intensive Care Department of Beijing Friendship Hospital (BFH) comprised 72 patients. Six machine learning models were employed in the prediction, namely the logistic, lasso, rpart, random forest, xgboost, and artificial neural network models. The comparative efficacy of the newly developed model in relation to the APACHE II model for predicting mortality risk was also assessed. The XGBoost model exhibited a superior performance with the training set. With the internal validation set and the two external validation sets (MIMIC-III and BFH), the xgboost algorithm demonstrated the highest performance. Meanwhile, APACHE II performed poorly at predicting the mortality risk with the BFH validation set. The mortality risk was influenced by three primary clinical parameters: urine volume, lactate, and Glasgow Coma Scale (GCS) score. Thus, we developed a prediction model for the risk of death among S-AKI patients that has an improved performance compared to previous models and is a potentially valuable tool for S-AKI prediction and treatment in the clinic.

## 1 Introduction

Sepsis is the term for life-threatening organ dysfunction caused by a dysregulated host response to infection. One complication of sepsis is sepsis-induced acute kidney injury (S-AKI), which is defined as a syndrome with the simultaneous presence of Sepsis 3 (2016) [1] and Kidney Disease Improving Global Outcomes (KDIGO; 2012) [2] criteria [3]. Approximately 33 % of the patients who are diagnosed with sepsis have acute kidney injury (AKI) [4]. In Intensive Care Units (ICUs), the incidence of AKI ranges from 40 % to 50 %, and the death rate due to AKI is about 40 % [4]. Approximately 11 % of fatalities occurring in hospitals can be attributed to untimely identification and intervention when the conditions are deteriorating [5]. Therefore, timely categorization of the prognosis of individuals is desired for early disease identification and treatment.

Machine learning has provided promising outcomes in numerous applications in healthcare, such as diagnosis, monitoring, treatment, medication discovery, and mortality risk forecasting [6]. It has demonstrated a superior performance in identification and predictions compared with conventional statistical approaches and novel biomarkers [7]. Moreover, machine learning is the preferred approach for intricate issues that entail noisy and inhomogeneous elements related to the outcome of prediction [8]. Multiple studies have attempted to leverage machine learning modeling for a more accurate prediction of risk of death for SAKI patients. However, only limited aspects of model performance have been considered, making it difficult to comprehensively compare the advantages and disadvantages of the models due to the imbalanced labels of existing clinical datasets. In addition, the majority of the models were not externally validated; thus, the wide applicability of the model also cannot be guaranteed [9]. Some models were developed using the AKI dataset for predicting the risk of death in SAKI patients, which is subject to bias in cohort selection. Furthermore, many studies did not calculate the sample size before constructing the final models [[10], [11], [12], [13]]. This issue easily leads to ineffectiveness of the model. A well-known rule of thumb for the sample size calculation is to ensure at least 10 events per candidate predictor parameter (EPPs). However, this rule might not be suitable for all models. Therefore, it has been suggested to increase the EPP value depending on the nature of the disease, including the number of events, the number of participants, the incidence rate, and the expected predictive performance.

This study aimed to develop high-performance machine learning models for death risk prediction for patients with S-AKI and to comprehensively evaluate their performance with both the internal dataset and multiple external datasets. Minimum sample size requirements were estimated for each dataset following the four-step procedure proposed by Riley et al. [14]. To fully evaluate the bias risk and potential effectiveness of the prediction model, this study followed the Transparent Reporting for Individual Prognosis or Diagnosis [15] during model development and performance evaluation, including the assessment of differentiation and calibration as well as decision curve analysis (DCA) [16]. The precise and reliable prognostic prediction from the resulting machine learning models can benefit both the patients and healthcare professionals so that they can implement timely and optimal decision-making for both the health and lives of patients.

## 2 Materials and methods

### 2.1 Datasets

In this study, the datasets came from Medical Information Mart for Intensive Care (MIMIC)-IV 1.0 [17], MIMIC-III Clinical Database 1.4 [18], and the Department of Critical Care at Beijing Friendship Hospital affiliated with Capital Medical University (BFH). MIMIC-III is a publicly available database. It contains over 40,000 unidentified patients, which were admitted to the ICU of the Beth Israel Deaconess Medical Center between 2001 and 2012. MIMIC-IV is an enhanced version of the MIMIC-III database. It contains samples captured from 2008 to 2019. It was used for both the training and internal validation of the machine learning models, while MIMIC-III was used for external validation. In addition, the samples collected from the Intensive Care Department of BFH are from S-AKI patients from December 2016 to January 2020. Both the MIMIC-III and BFH datasets were used for external validation.

### 2.2 Study population

The inclusion criteria for this study were as follows: 1. patients with S-AKI meeting both Sepsis-3.0 (2016) and KDIGO (2012) AKI standards; 2. patients were in their first stay of the ICU during hospitalization; and 3. S-AKI occurred on the day of check-in to the ICU, specifically between 6 h before and 24 h after the ICU admission time. The exclusion criteria for this study were as follows: 1. patients aged less than 18 years old or greater than 89 years old; and 2. patients who stayed in the ICU for less than 24 h.

According to the Sepsis 3.0 standard, we defined sepsis as a rapid increase in sequential organ failure assessment (SOFA) score ≥2 after suspected infection. The suspected infection was defined as the use of antibiotics between 24 h before and 72 h after body fluid culture [19]. For the BFH dataset, both sepsis and AKI were confirmed according to the clinical diagnosis record. For the patients who entered the ICU multiple times during a single hospitalization, only the first admission to the ICU was analyzed. The same inclusion and exclusion criteria were applied to both the MIMIC-III and BFH datasets. After the processing of MIMIC-IV samples with missing items, 70 % of these cases were incorporated into the training set, while the other 30 % of cases were employed as the internal validation set.

### 2.3 Outcomes and predictor variables

The main outcome measure was Hospital_expire_flag, which is a binary flag indicating whether the patient died within the given length of hospital stay. Specifically, 1 represents death in the hospital, and 0 represents survival until discharge.

The predictive variables consisted of demographic indicators (age, sex, marital status, race, height, and weight), vital signs (body temperature, heart rate, heart rhythm, respiratory rate, systolic blood pressure, diastolic blood pressure, mean arterial pressure, pulse oxygen saturation, and urine volume), laboratory test results (blood gas analysis and total oxygen CO_2_, blood cytology, blood electrolytes, blood albumin, globulin, A1C glycosylated hemoglobin, anion gap, renal function indicators, coagulation indicators, liver function indicators, and myocardial enzymology indicators). Among them, the diagnosis (the top 20 diagnoses with the highest incidence rate), extreme values of treatment items (blood filtration, blood filtration mode, antibiotic type, vasopressor drugs, mechanical ventilation method), and severity scores (AKI grade, SOFA score, and Glasgow Coma Scale (GCS) score) were taken as the candidate variables for screening the predictive variables. Referring to the work of Segar et al. [20], the covariates missing ≥20 % and the covariates with a correlation coefficient ≥0.70 were excluded.

### 2.4 Sample size

The sample size was calculated using the method proposed in the work of Riley et al. [14]:

Step 1: Calculate the sample size N1 required to evaluate the overall outcome risk or average outcome value. Calculation formula: (1.96/δ)^2*Φ*(1–Φ), where δ is the absolute error range, usually ≤0.05; and Φ is the predicted proportion of outcomes. In this research, δ = 0.05 and Φ = 0.22144. Thus, N1 = 264.93.

Step 2: Calculate the sample size N2 for generating the predicted value with the least mean error for all individuals. Calculation formula: exp((−0.508 + 0.259*log(Φ)+0.504 *log(p)–log(MAPE))/0.544), where p is the number of candidate prediction parameters; Ф is the expected result ratio (≤0.5); and MAPE is the average absolute prediction error, usually ≤0.05. In this study, Φ = 0.22144, p = 42, and MAPE = 0.05. Thus, N2 = 1507.4.

Step 3: Calculate the sample size N3 for guaranteeing relatively small shrinkage. Calculation formula: p/((s–1)*log(1–R^2^/s)), where p is the number of candidate prediction parameters; s is the shrinkage surplus, usually with a shrinkage rate of ≤0.1, i.e., s ≥ 0.9; and R^2^ is the determination coefficient. For death risk prediction, R^2^ = 0.5*maxR^2^, maxR^2^ = 1-exp (2*L/n), and L = E*log (E/n)+(n-E)*log(1–E/n). Among them, *E* represents the total number of people with outcomes, and n denotes the sample size. In this study, p = 42, s = 0.9, E = 886, n = 4001, L = −2115.5, maxR^2^ = 0.65266, and R^2^ = 0. 32,633. Thus, N3 = 932.62.

Step 4: Calculate the sample size N4 for improving the performance of model fitting. Calculation formula: p/((s–1)*log(1–R^2^/s)), where p is the number of candidate prediction parameters; s = R^2^/(R^2^+δ*maxR^2^); R^2^ is the determination coefficient; and δ is the absolute error range, usually ≤0.05. In this study, p = 42, δ = 0.05, and R^2^ = 0.32633. Thus, N4 = 1039.

According to the above process, the sample size required in this study should be greater than 1507.4, which is 1508 samples.

### 2.5 Screening process

**Fig. 1:**
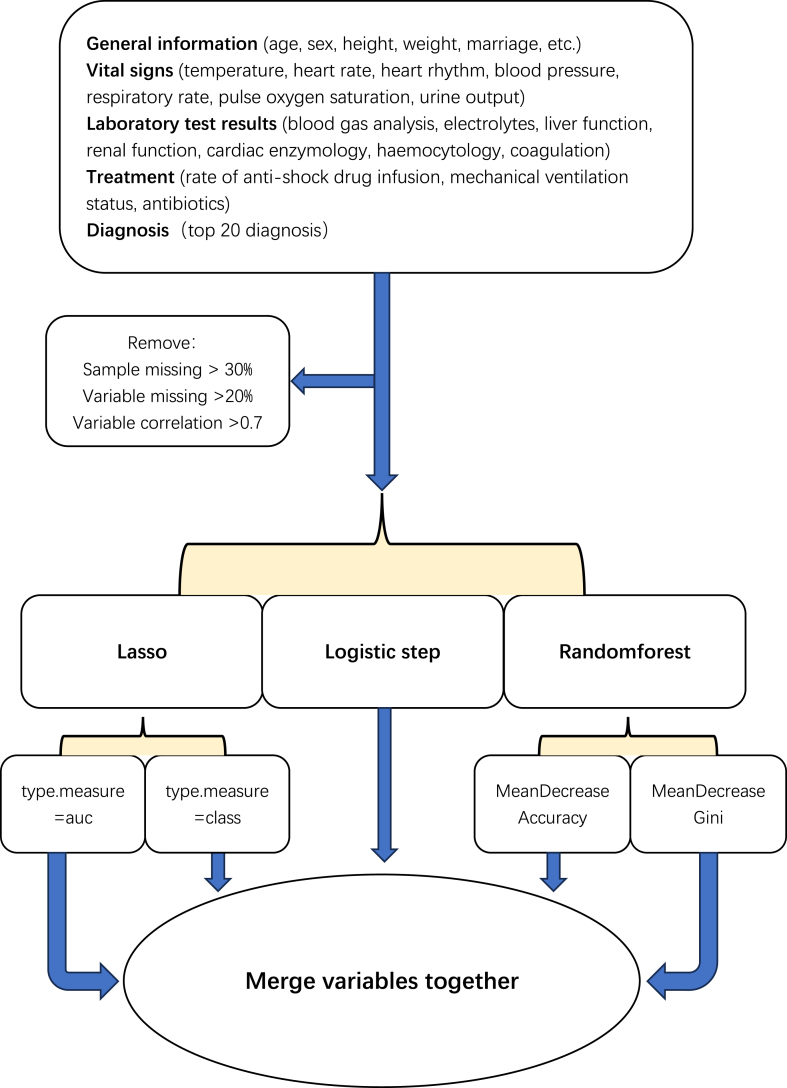
The entire screening process used in this study.

For predictive factor screening, the software used for data organization in this study included postgreSQL13, pgAdmin4, R (4.2.2), and Rstudio for Windows 2022.12.0. The generated variables included general information of the patients (age, sex, height, weight, marriage, etc.), first day vital signs (body temperature, heart rate, heart rhythm, blood pressure, respiratory rate, pulse oxygen saturation, and urine volume), first day blood gas analysis results (total oxygen CO_2_ calculated based on the first day blood gas analysis and hemoglobin), laboratory test results on the first day (electrolytes, liver function indicators, renal function indicators, myocardial enzymology indicators, blood cytology indicators, and coagulation indicators), diagnosis (the top 20 diagnoses with the highest incidence rate), and treatment (vasopressor drugs, mechanical ventilation status, and antibiotics). Of note, the multiclass variables were converted into virtual variables. The samples with a missing ratio of ≥30 % were removed, and the covariates with missing ratios of ≥20 % were also deleted. In addition, the correlation analysis was performed on numerical variables, and the variables with a correlation greater than 70 % were deleted. Three methods were used for variable screening: 1. logistic stepwise regression, using the Akaike information criterion standard and direction = “both,” the variables with *p* < 0.05 were generated; 2. lasso regression, using the area under the receiver operating characteristic curve (AUROC) and the misclassification error for calculating the cross-validation losses, a 10-fold cross-validation scheme was adopted to determine the maximal lambda value while minimizing the cross-validation error within a range of 1 standard deviation, and the variables with regression coefficients other than 0 were generated; and 3. random forest, to obtain the number of variables with a minimum error rate, the 10-fold cross-validation was employed. To obtain the valuable variables, the features were ranked using the Gini index and the information gain method. Variables were obtained independently using each of these methods and merged to yield the final variables for model development. The entire screening process is shown in Fig. 1.

### 2.6 Model construction

With the selected feature variables, six methods were employed to construct the prediction model: 1. For the logistic regression algorithm, the glm function in the R stats package was used to construct the model. 2. For the lasso algorithm, the glmnet function in the R glmnet package was used to build the model, with 100 different lambda values, and penalty term alpha = 1.3. The rpart algorithm used the rpart function in the R rpart package to build the model, with the method of “class” and the splitting index of Gini. According to the complexity parameter corresponding to the minimum cross-validation error “xerror,” the prune function was used to obtain this model. 4. The randomForest function in the R randomForest package was used to construct the model. At each split, a subset of variables was randomly sampled as candidates. The number of candidates was determined as the square root of the default number of variables in the model. The number of growing trees was initially set to 500 and optimized by the lowest prediction error. After parameter optimization, the maximum number of leaf nodes (maxnodes) was set to 30 and the sample size for bootstrapping (sampsize) was set to 50 %. 5. For xgboost, the expand. grid function in the caret package was first used to determine the adjustment parameter grid, including the number of iterations, maximum depth of a tree, learning rate, minimum segmentation loss, proportion of subsamples to construct each tree, total minimum instance weights required for subinstances, and the subsample ratio of the training instance. Next, the best parameters were tuned using the 10-fold cross-validation method in the train function. Finally, the xgb. train function in the xgboost package was used to train the model. 6. For ANN, the data were normalized using the preProcess function in the caret package. The neuralnet function in the neuralnet package was used to construct the model. All six models were stored in RData files, and individuals could be predicted by adopting a single sample variable in R. The prediction statements in the development model package were used for the individuals. The R packages used included stats, glmnet, rpart, randomForest, xgboost, and neuralnet.

### 2.7 Performance evaluation and validation

Generally, this study used the bootstrap algorithm to evaluate the performance of the six algorithms mentioned above. In addition, the performance of the models was evaluated in terms of three dimensions: discrimination, consistency, and clinical effectiveness. In total, the performance indicators included ROC, PRC, AUROC, f1score, youden_Index, and IDI. The calibration indicators included calibration curve, Brier_score, and kappa coefficient. DCA was the clinical applicability indicator. Seventy percent of the MIMIC-IV dataset was used as the training set, and the other 30 % was used as the internal validation set. The MIMIC-III data and the BFH data were used as the external validation sets.

### 2.8 Statistical analysis

PostgreSQL13 and pgAdmin4 were used to manage the databases. R software (4.3.1) was employed for statistical analysis, and R studio software 2022.12.0 Build 353 was used for visualization. Continuous variables were represented as the median with inter-quartile spacing or the mean with standard deviation. Meanwhile, the categorical variables were represented by the frequency and percentage. The Shapiro–Wilk test was used to evaluate the normality of the distribution for continuous variables. Additionally, the classification variables were compared using the chi-squared test or Fisher's test. Non-normal continuous variables were compared using the Kruskal–Wallis rank sum test. A value of *p* < 0.05 was considered as statistically significant.

## 3 Results

### 3.1 Baseline characteristics and outcomes of the participants

**Fig. 2:**
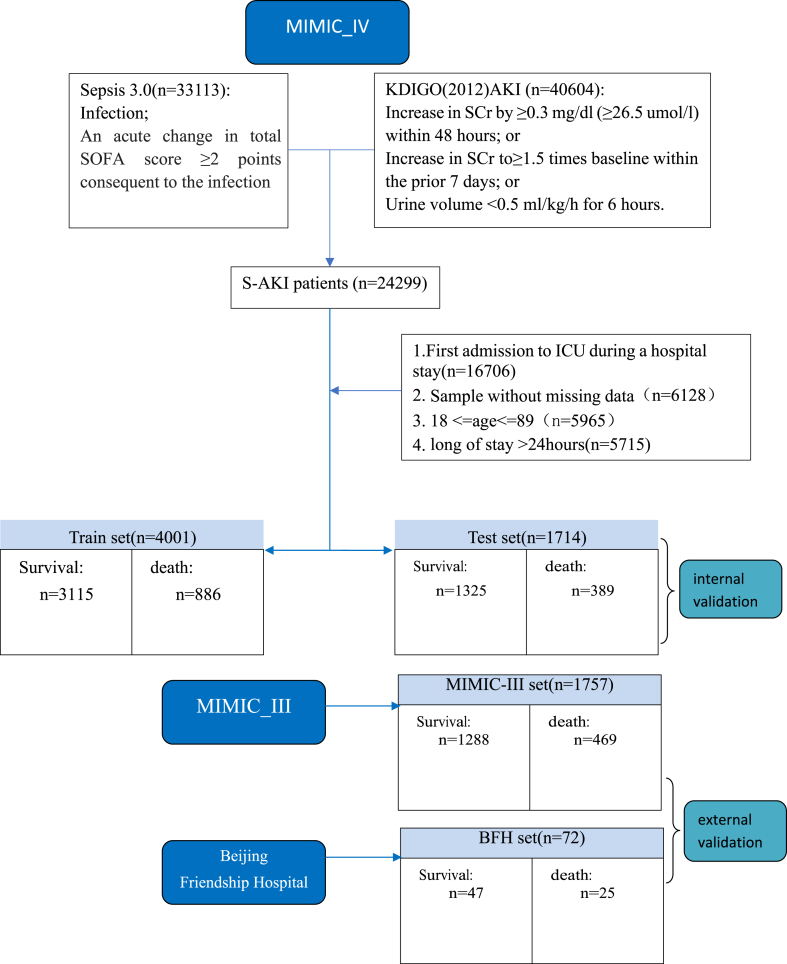
The process of sorting the participants in this study.

Among the 382,278 samples in the MIMIC-IV database, 33,113 samples of sepsis 3 and 40,604 samples of AKI were exported, and 24,299 S-AKI samples were obtained with coexistence of sepsis and AKI. The hospitalization mortality rates of sepsis patients, AKI patients, and S-AKI patients were 15.6 %, 14.7 %, and 19.2 %, respectively. Finally, 5715 samples of the MIMIC-IV dataset were selected after excluding samples with missing values and applying the rest of the inclusion and exclusion criteria, with 4001 samples assigned to the training set and 1714 to the interval validation set. The number of training samples surpassed the minimum requirement (1508) from the sample size calculation. In general, the missing rate was 7.2 %. The maximum missing proportion for one variable was 46.9 %, and the missing proportion of samples was 61.9 % at most. Meanwhile, 1757 samples in the MIMIC-III dataset and 72 samples in the BFH dataset were selected as the external validation sets. The process of sorting the participants to be included in this study is depicted in Fig. 2, and the baseline characteristics of the participants are provided in Supplementary Table 1.

### 3.2 Model development

**Fig. 3:**
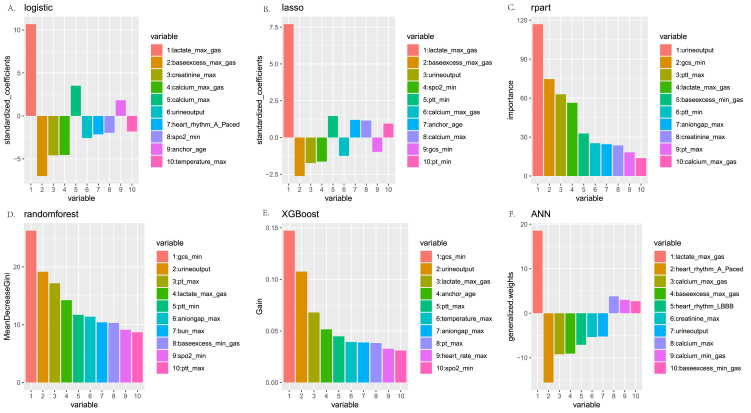
The top ten variables used in the six models. The panels in the figure are (A) the top variables in the regression coefficients of the logistic model, (B) the variables in the regression coefficients of the lasso model, (C) the variables used in the decision tree model, (D) the variables of importance in the random forest model, (E) the variables of importance in the XGBoost model, and (F) the top five variables in the generalized weights of the ANN model.

During variable selection, 31 variables were selected by logistic stepwise regression, 29 and 37 variables were selected by lasso using AUC and accuracy as scores, respectively, and 32 and 32 variables were selected by the random forest model using the Gini index and accuracy, resulting in 42 variables after merging. The contributions of various variables in the model were analyzed. The top five variables in the regression coefficients of the logistic model included the maximum lactate value, maximum alkaline residue value, maximum creatinine value, maximum blood gas calcium value, and maximum blood calcium value. The top five variables in the regression coefficients of the lasso model were the maximum lactate value, maximum alkaline residue value, first day urine output, pulse oxygen saturation, and partial thromboplastin time. The variables used in the decision tree of the Rpart model were the first day urine output, minimum GCS value, partial oxygen pressure, partial thromboplastin time, maximum lactate value, maximum prothrombin time, and age. The top five variables of importance in the random forest model were lowest GCS score, the first day urine output, maximum prothrombin time, maximum lactate value, and minimum partial thromboplastin time. The top five variables of importance in the XGBoost model were the lowest GCS score, urine output, maximum lactate value, age, and maximum partial thromboplastin time. The top five variables in the generalized weights of the ANN model were the maximum lactate value, atrial tachycardia, maximum blood gas calcium value, maximum alkaline residue value, and left bundle branch block, as shown in Fig. 3.

### 3.3 Model performance

**Fig. 4:**
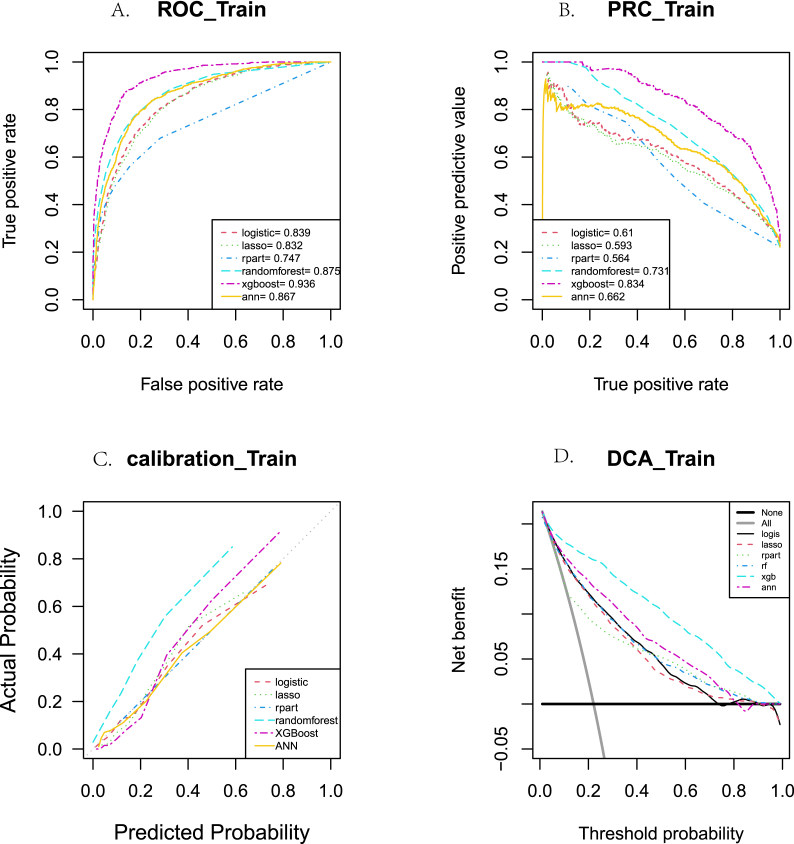
The differences between the six models in terms of receiver operating characteristic (ROC) curve analysis (A), precision-recall curve (PRC) analysis(B), calibration curve (C), and decision curve analysis (DCA) (D) using the training set.

Using the training set, the performance of each model is provided in Fig. 4 and Supplementary Figs. 1–2. Among them, the XGBoost model performed the best, with AUROC = 0.937 (0.932, 0.941), AUPRC = 0.834 (0.824, 0.845), F1 score = 0.743 (0.732, 0.753), youden_Index = 0.738 (0.725, 0.750), Brier_score = 0.084 (0.082, 0.087), and kappa = 0.655 (0.642, 0.667). The IDI, ROC, PRC, calibration curve, and DCA curve also demonstrated that the XGBoost model outperformed the competing models.

**Fig. 5:**
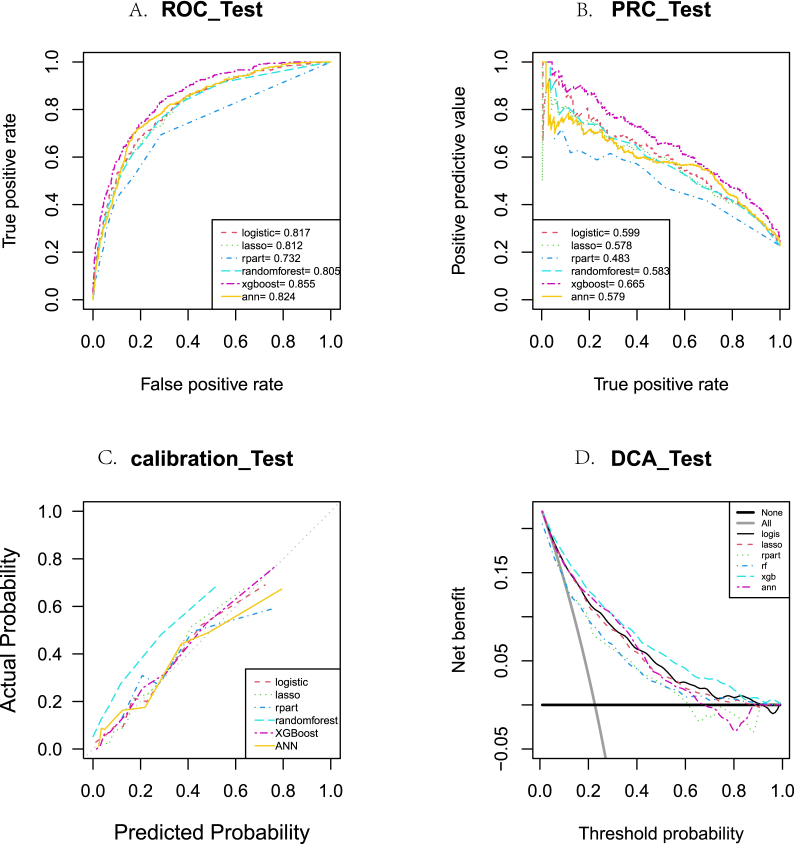
The differences between the six models in terms of (A) receiver operating characteristic (ROC) curve analysis, (B) precision-recall curve (PRC) analysis, (C) calibration curve, and (D) decision curve analysis (DCA) in the internal validation.

The performance of each model using the validation set is provided in Fig. 5 and Supplementary Figs. 3–4. Among all of the indicators, the XGBoost model achieved the optimal performance, with AUROC = 0.856 (0.846, 0.866), AUPRC = 0.665 (0.642, 0.687), f1score = 0.613 (0.595, 0.631), youden_index = 0.536 (0.511, 0.560), kappa value = 0.477 (0.455, 0.499), and Brier_score = 0.119 (0.114, 0.124). In addition, XGBoost also achieved the best results in terms of IDI, ROC, PRC, calibration curve, and DCA.

The performance of the six models using the external validation set MIMIC-III is shown in Supplementary Figs. 5–7. The XGBoost model performed the best, with AUROC = 0.784 (0.772, 0.796), AUPRC = 0.604 (0.582, 0.626), and Brier_score = 0.154 (0.148, 0.160). Meanwhile, the XGBoost model also achieved optimal performance in terms of IDI, ROC, PRC, calibration curve, and DCA.

**Fig. 6:**
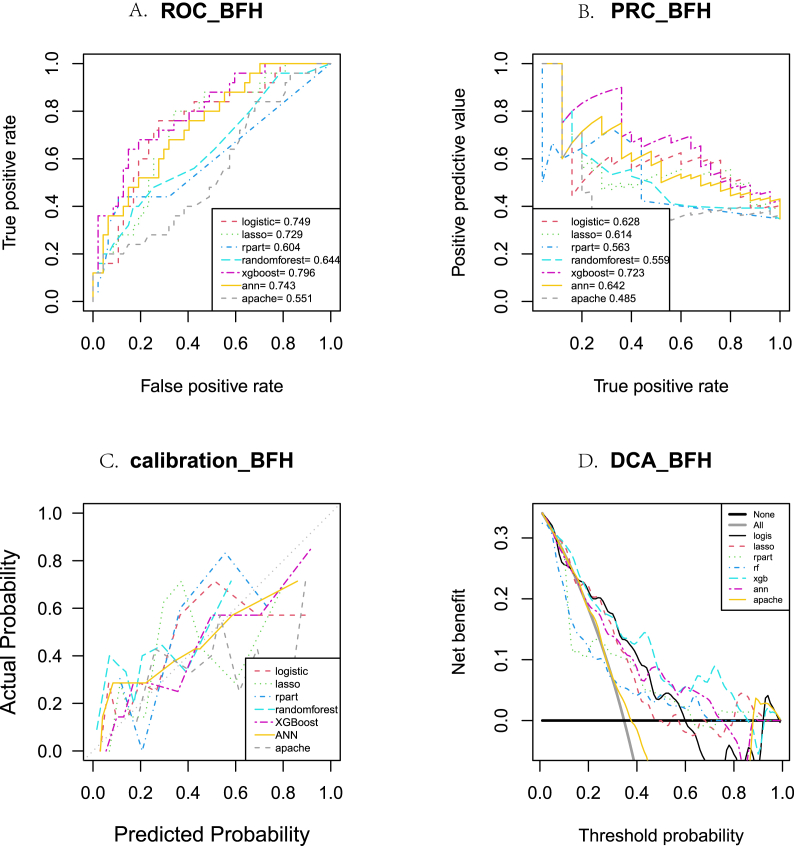
The differences between the six models in terms of (A) receiver operating characteristic (ROC) curve analysis, (B) precision-recall curve (PRC) analysis, (C) calibration curve, and (D) decision curve analysis (DCA) using the validation set from Beijing Friendship Hospital (BFH).

The performance of each model using the validation set BFH is shown in Fig. 6 and Supplementary Figs. 8–9. Among the six models, the XGBoost model was the most outstanding, with AUROC = 0.797 (0.742, 0.852), AUPRC = 0.719 (0.628, 0.811), and Brier_score = 0.172 (0.147, 0.197). In addition, the XGBoost model achieved the optimal performance in terms of IDI, ROC, PRC, calibration curve, and DCA. Compared with the APACHE II [21] model, the presented model demonstrated superior performance.

## 4 Discussion

In this study, machine learning models for death prediction of S-AKI patients were developed from the MIMIC-IV dataset using six classifiers. These models were evaluated in three dimensions using nine indicators. They were also validated using three validation sets (one internal validation from MIMIC-IV and two external validations from the MIMIC-III and BFH datasets). The XGBoost model performed the best using the internal validation, with an AUROC of 0.856 (0.846, 0.866). The XGBoost model performed the best using the external validation dataset MIMIC-III, with an AUROC of 0.784 (0.772, 0.796). In addition, the AUROC of the best-performing XGBoost model in the external validation set BFH was 0.797 (0.742, 0.852). This result is an improvement over previous research [10,22,23].

The incidence of AKI is high in sepsis patients, and the incidence of AKI caused by sepsis is also high in AKI patients. In a substudy of a global multi-center audit [24], the incidence of AKI in sepsis patients was 68 %, and the hospitalization mortality rate of AKI patients with sepsis was 37.7 % greater than that of AKI patients without sepsis (25.9 %). Sepsis accounted for 27.9 % of AKI patients, and the hospitalization mortality rate of S-AKI patients was 17.1 % greater than that of the AKI patients without sepsis. As S-AKI has a high mortality rate, prognosis prediction plays a positive role in early diagnosis and timely treatment to reduce mortality.

Although it is generally believed that AKI is caused by sepsis in half of patients, it has been shown previously [25] that sepsis was responsible for only 23.6 % of AKI in affected patients. Therefore, AKI cannot be equated with S-AKI. To predict the risk of death in S-AKI patients, a prediction model needs to be developed. Of note, the prediction model developed in this study was based on patients with S-AKI.

Using the MIMIC database and machine learning methods, various patient death prediction models have been developed. For instance, Lin et al. [22] compared four models, SAPS-II, SVM, ANN, and random forest, using the MIMIC-III database, and confirmed that the random forest model had a better predictive performance on mortality rates in AKI patients than the others. Meanwhile, Huang et al. [23] developed a prediction model for AKI patients using the MIMIC-III database. They validated the proposed model using the eICU database and samples from Shenzhen Second People's Hospital. In addition, the random forest algorithm was proved to perform better than logistic regression models using SAPS II, APACHE IV, and SOFA. Zhou et al. [13] also developed multiple machine learning models for mortality prediction of S-AKI patients using the MIMIC-IV dataset, and the CatBoost model was reported to perform the best. However, only model discriminability was assessed during performance evaluation, and no sample size calculation was performed to justify the sufficiency of the training sample size. Our study employed a more comprehensive set of evaluation metrics, including the kappa coefficient, Brier score, IDI, AUPRC, and DCA, and the sample size calculation was included. In addition, different from simply removing the missing values in this study, the KNN algorithm was employed to impute the missing values. KNN imputation can be sensitive to noise and outliers as well as be susceptible to the choice of hyperparameters. As a multiple imputation method, it also suffers from low reliability and the possibility of losing significant features. Moreover, Luo et al. [10] developed the XGBoost prediction model for S-AKI patients using the MIMIC-IV and eICU databases and validated this model on both internal and external validation sets. The AUC of the internal test set was between 0.848 and 0.804, while the AUC of the external test set was between 0.818 and 0.748. In our study, a model was also developed using the MIMIC database. The AUC values of our proposed model were higher in both the internal validation (0.856 (0.846, 0.866)) and the external validation (0.797 (0.742, 0.852)).

The three most important predictive factors for the best-performing models in this study were urine volume, GCS score, and lactate. In the future, it may be possible to reduce mortality by taking these variables into consideration in clinical practice. Previous studies have suggested that biomarkers such as the fibrinogen-to-albumin ratio, urinary protein, urinary albumin, glycated hemoglobin, neutrophil gelatinase-associated lipocalin, kidney injury molecule-1, liver-type fatty acid-binding protein [25], and other indicators can be used to predict AKI mortality accurately. Additionally, Yoo et al. [26] found that the recurrent neural network model is better than classical methods for predicting the mortality of AKI patients with continuous renal replacement therapy and improves treatment outcomes by improving important predictive factors. In the compilation process, different attempts were made by us. We attempted to include antibodies against hepatitis A, B, and C antigens as well as 32 urinary laboratory tests including urinary protein, urinary albumin, urinary creatinine, urinary sodium, urinary glucose, glycated hemoglobin A1C, arterial oxygen levels, troponin I, troponin T, and B-type natriuretic peptide. However, they were removed due to excessive deletions or filtered out during variable screening. Once a tidier dataset can be obtained, current models can be further improved using these variables.

To evaluate the predictive performance of a machine learning model, we also need to compare whether one of the two models can predict risks more accurately [27]. The datasets of medical research mostly consist of imbalanced data, and most machine learning methods are sensitive to imbalanced data [28]. Therefore, a single evaluation indicator usually is insufficient to evaluate the model performance. Some studies have used single evaluation indicators. However, these single indicators can only reflect sensitivity and specificity. In addition, they cannot be taken to reflect the constraints of sensitivity on precision. The lack of calibration and clinical applicability will further reduce the comprehensiveness of efficacy evaluation.

In previous studies, the AUROC of externally validated prediction models was usually low (less than 0.7). Meanwhile, the AUROC of internal evaluation was always lower than the external validation [29]. Similar to these studies, the current study also achieved a better outcome in the internal validation. The XGBoost model performed the best in the internal validation and in both of the external validations. This differs from the results of some studies, where Huang et al. [23] and Lin et al. [22] have confirmed that the random forest model is the optimal model using the MIMIC-III dataset. They also found that the clinical applicability of the random forest model using the MIMIC-III database was optimal. However, in our study, the xgboost model was the best-performing model in terms of discrimination and calibration as well as clinical applications. In the work of Wilson et al. [7], the quality of data samples was determined to be more important than the adoption of algorithms for predictive performance. Therefore, the solution to improve the performance of the models might lie in the data collection process. Accordingly, institutions can try to employ historical data for establishing prediction models, and the models can be further used to guide decision-making in clinical cases. This is a potentially valuable way to solve the model efficiency problem, which conforms to precision medicine. Moreover, a database with global data should be established. Then, a prediction model can be developed using this database for improving clinical applicability.

This study also has several limitations that must be addressed. First, the patients in the dataset used were aged between 18 and 89 years old. Therefore, the application of the model is limited to only those in this age range. Second, the dataset used was imbalanced, which is not conducive to the development and evaluation of the model. Third, the proposed model was built upon the data from the first day of admission to the ICU, which lacks the subsequent data changes on the outcomes. Fourth, this study removed a large number of training samples with missing items, which may have resulted in the loss of information and increased the risk of overfitting. Fifth, although the independent testing dataset MIMIC-III, which was collected from the same institution as the training dataset MIMIC-IV, had a large sample size of 1757, the number of patients in the other testing dataset BFH was limited. This may have reduced the credibility of the external validation performance of the developed models and increased the risk of overfitting across different institutions. Finally, the presented model relies on R software for visualization and application, which might not be friendly for non-R software users.

## 5 Conclusion

In this study, we developed a prediction model for the risk of death among S-AKI patients using various machine learning algorithms that has an improved performance compared to previous models. Therefore, the proposed model is a potentially valuable tool for S-AKI prediction and treatment in the clinic.

## Data availability statement

The MIMIC-IV and MIMIC-III datasets analyzed in this study can be found online at https://mimic.mit.edu/. Lei Dong completed registration on PhysioNet and became an authenticated user. The training course for human subject research was completed, with certificate number 39791567. The Data Usage Agreement (DUA) was also signed.

## Ethics statement

This study was conducted after the approval of the Bioethics Committee of Beijing Friendship Hospital Affiliated to Capital Medical University, in accordance with the ethical principles of the Opinions of the General Office of the Communist Party of the China Central Committee and the General Office of the State Council on Deepening the Reform of the Evaluation and Approval System and Encouraging Innovation in Pharmaceutical and Medical Devices (2017), the Measures for Ethical Review of Biolife Research Involving Human Beings (2016) issued by the National Health and Family Planning Commission, the National Drug Administration guidelines of the National Health Commission's “Quality Management Standards for Clinical Trials of Drugs” (2020), the National Health and Family Planning Commission's “Quality Management Standards for Clinical Trials of Medical Devices” (2016), the World Medical Association's “Helsinki Declaration,” and the Council for International Organizations of Medical Sciences' “International Ethical Guidelines for Human Biolife Research.” This project was approved by the Academic Committee of Beijing Friendship Hospital Affiliated to Capital Medical University, with project approval number BFH20230322001 and research number BFHHZS20230094, and recorded in the Medical Research Registration and Filing Information System of the National Health Security Information Platform, Medical Research, with filing number MR-11-23-020,147.

## CRediT authorship contribution statement

**Lei Dong:** Writing – review & editing, Supervision, Software, Investigation, Data curation, Conceptualization. **Pei Liu:** Writing – original draft, Methodology, Funding acquisition, Conceptualization. **Zhili Qi:** Writing – review & editing, Resources, Funding acquisition, Data curation. **Jin Lin:** Writing – review & editing, Supervision, Project administration, Data curation. **Meili Duan:** Writing – review & editing, Validation, Project administration, Data curation.

## Declaration of competing interest

The authors declare that they have no known competing financial interests or personal relationships that could have appeared to influence the work reported in this paper.
