# Supplementary figures and images for: Development and validation of a machine-learning model for predicting the risk of death in sepsis patients with acute kidney injury
Source: Heliyon. 2024 Apr 20;10(9):e29985. doi: 10.1016/j.heliyon.2024.e29985 (PMC11064448; doi:10.1016/j.heliyon.2024.e29985)

### AUROC\_Train

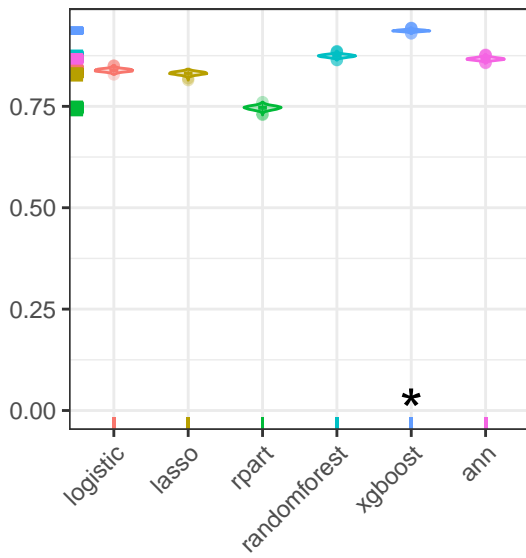

### AUPRC\_Train

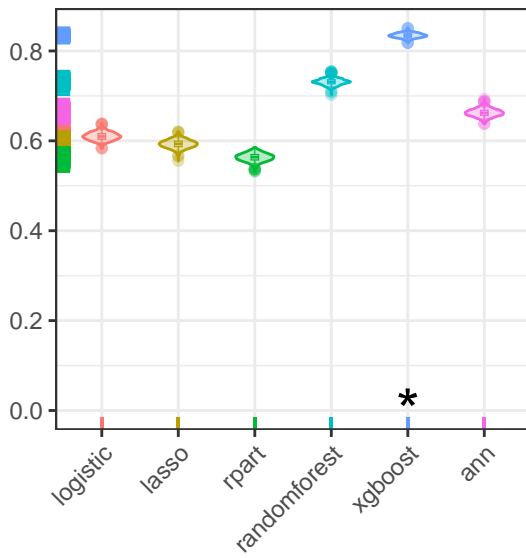

### f1Score\_Train

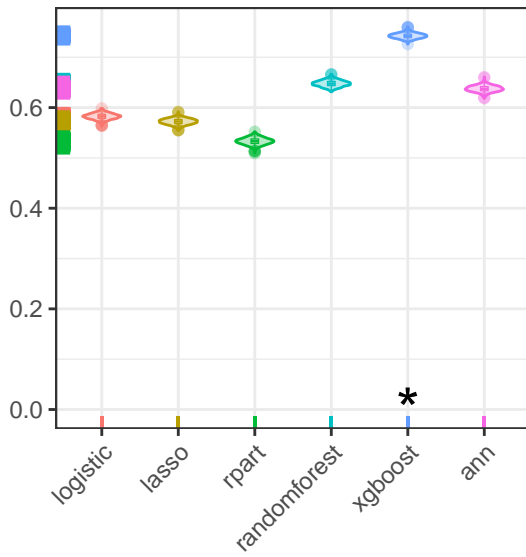

### youden\_Train

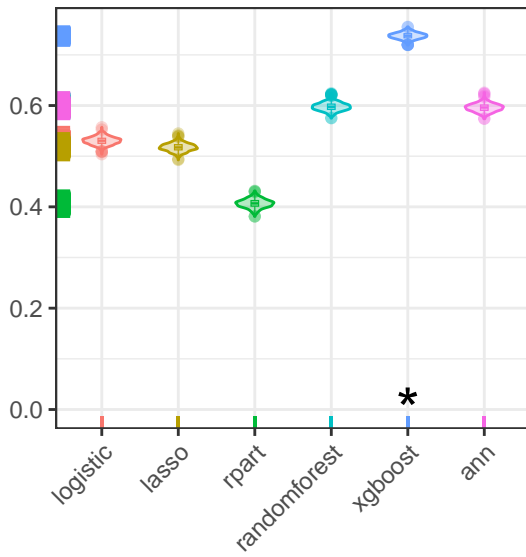

Supplement: Multimedia component 1 [file mmc1.pdf]

Brier\_Train

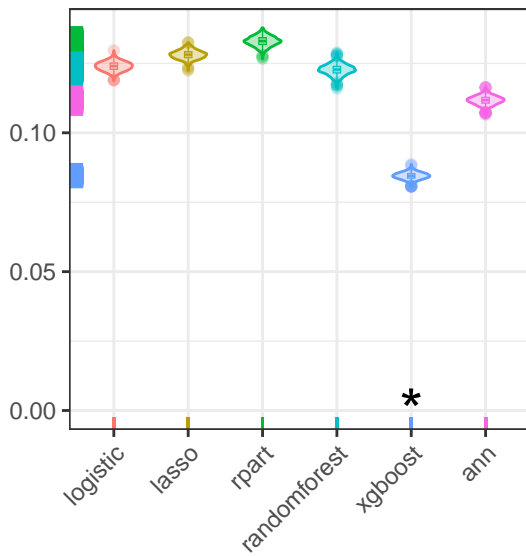

kappa\_Train

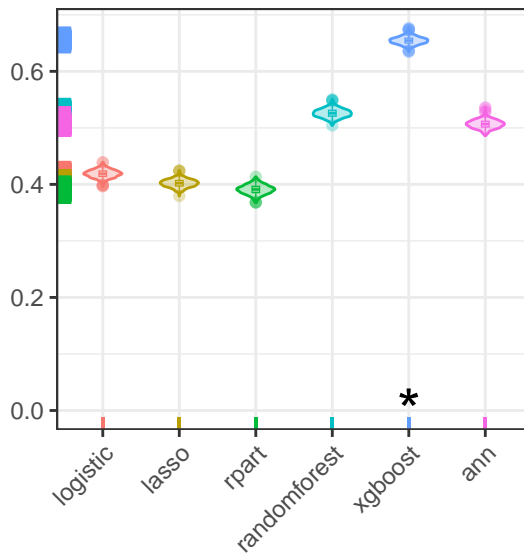

IDI\_Train

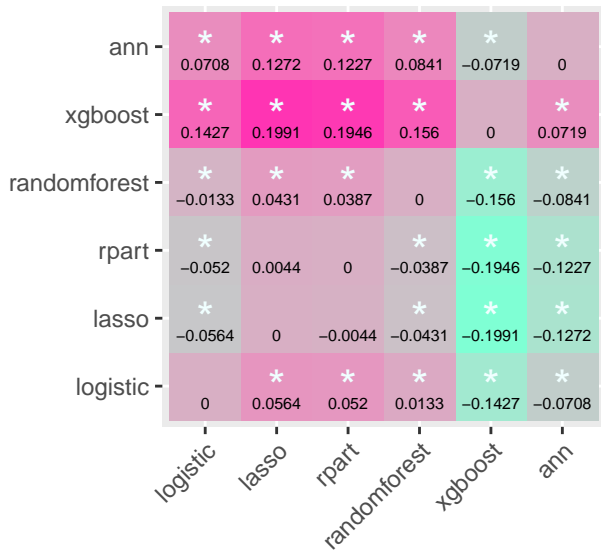

roc.test\_Train

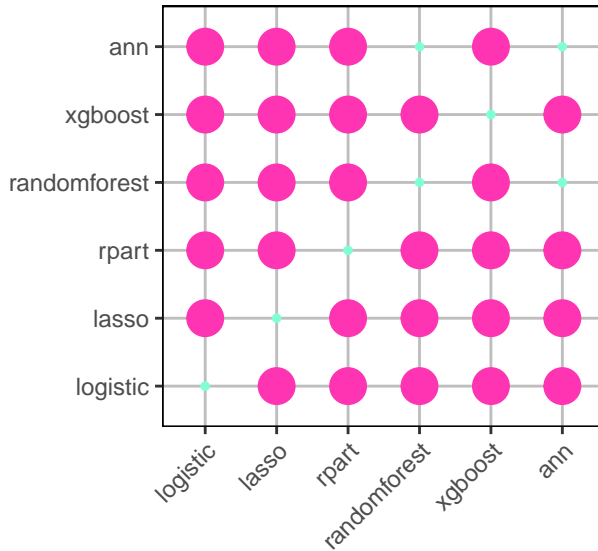

Supplement: Multimedia component 2 [file mmc2.pdf]

AUROC\_Test

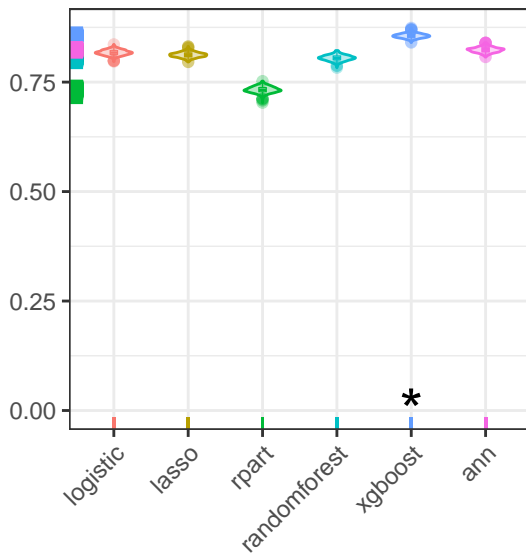

AUPRC\_Test

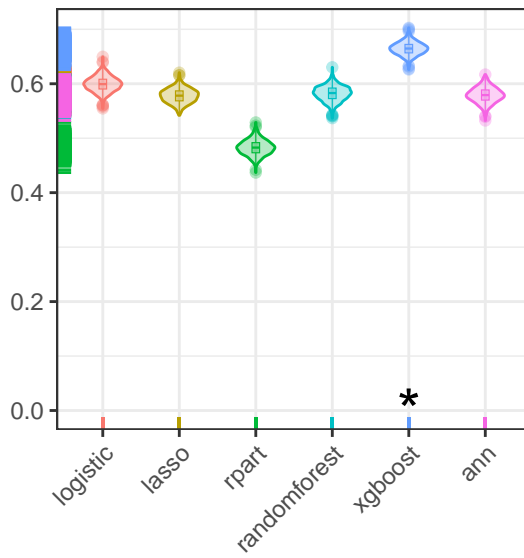

f1Score\_Test

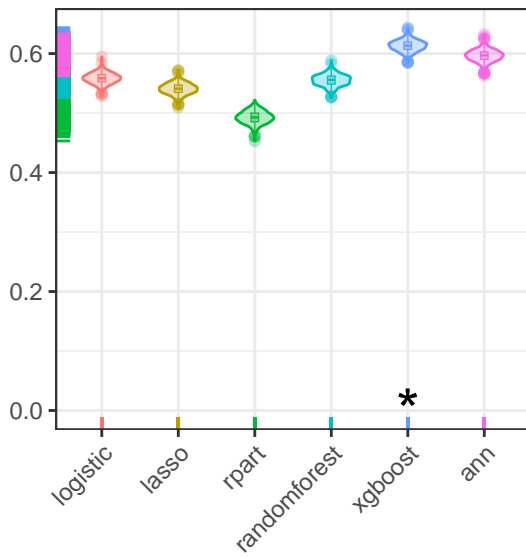

youden\_Test

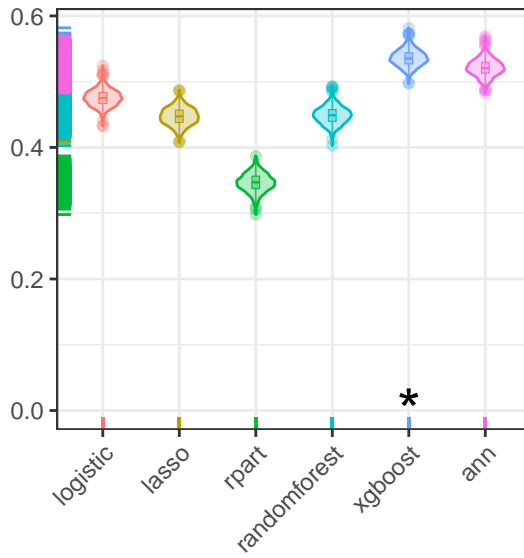

Supplement: Multimedia component 3 [file mmc3.pdf]

Brier\_Test

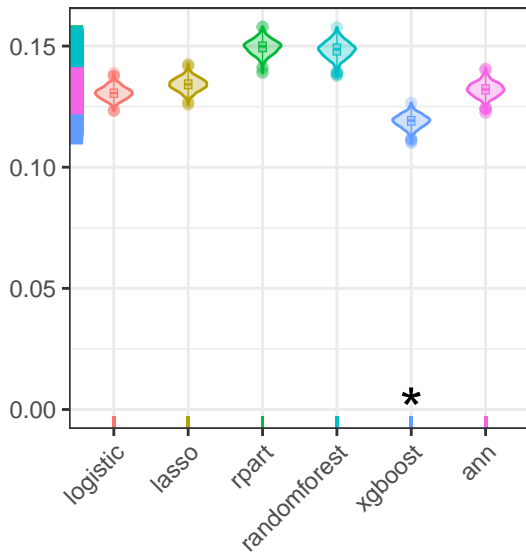

kappa\_Test

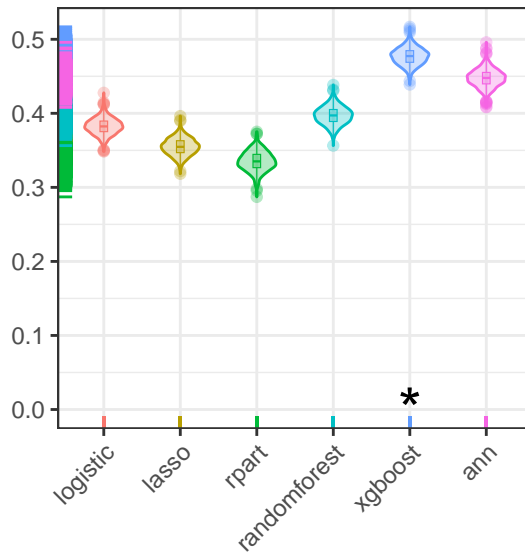

IDI\_Test

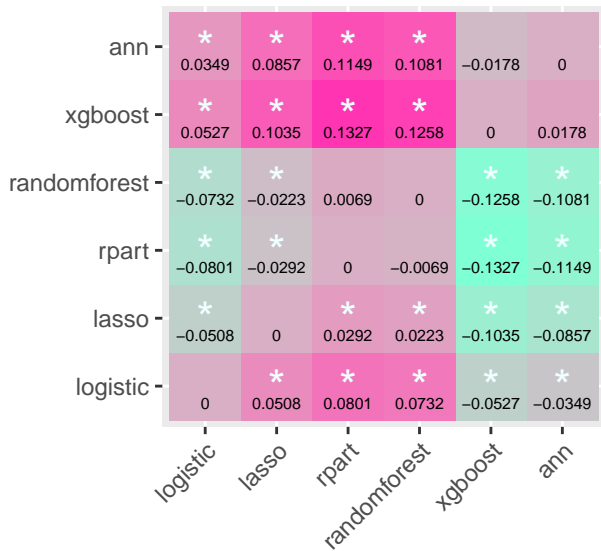

roc.test\_Test

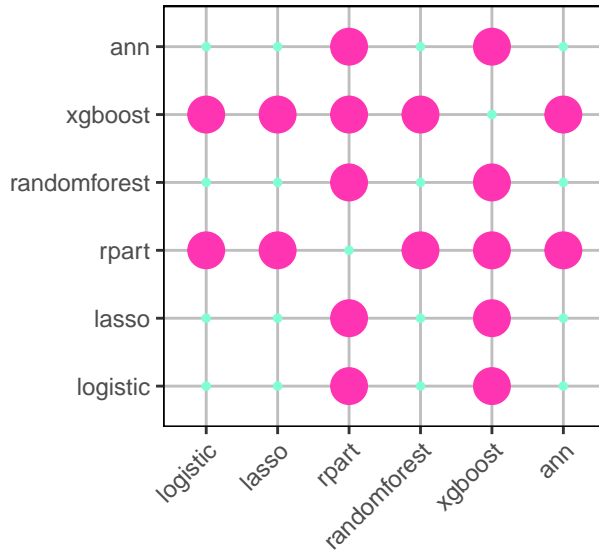

Supplement: Multimedia component 4 [file mmc4.pdf]

### ROC\_MIMIC-III

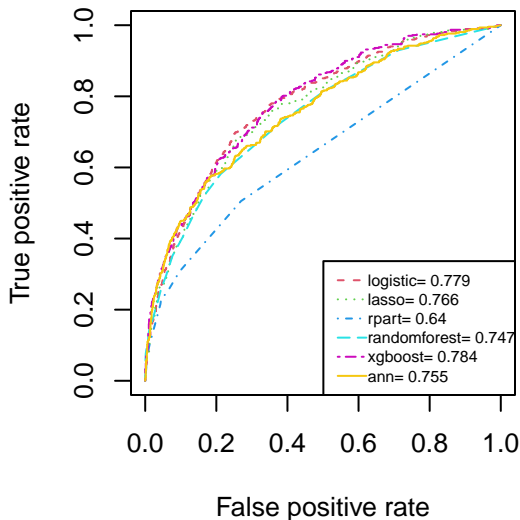

### PRC\_MIMIC-III

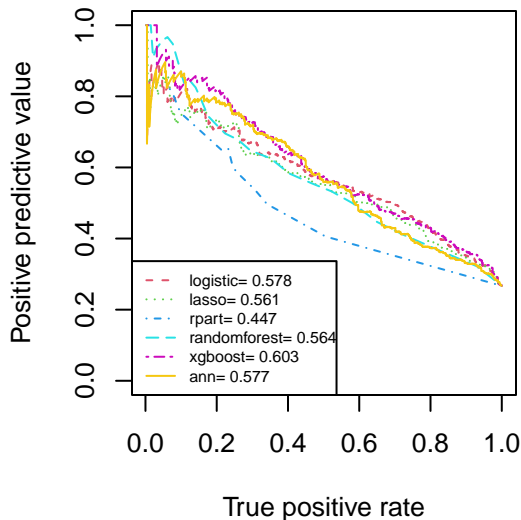

### calibration\_MIMIC-III

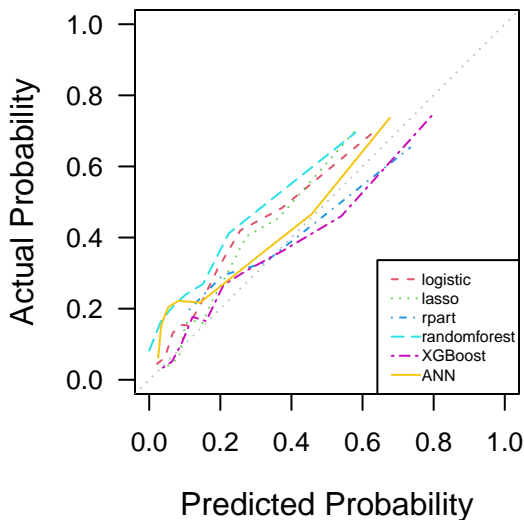

### DCA\_MIMIC-III

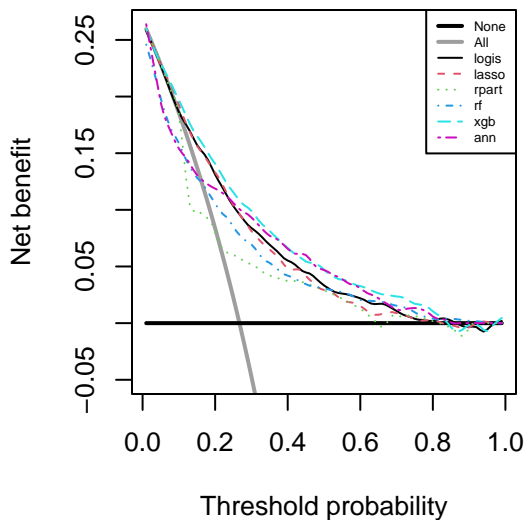

Supplement: Multimedia component 5 [file mmc5.pdf]

AUROC\_MIMIC-III

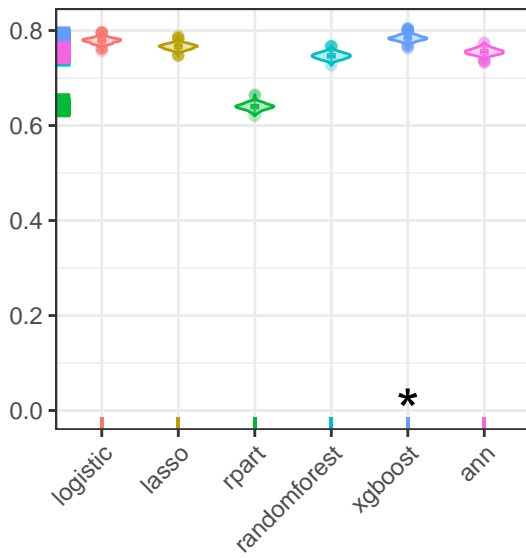

AUPRC\_MIMIC-III

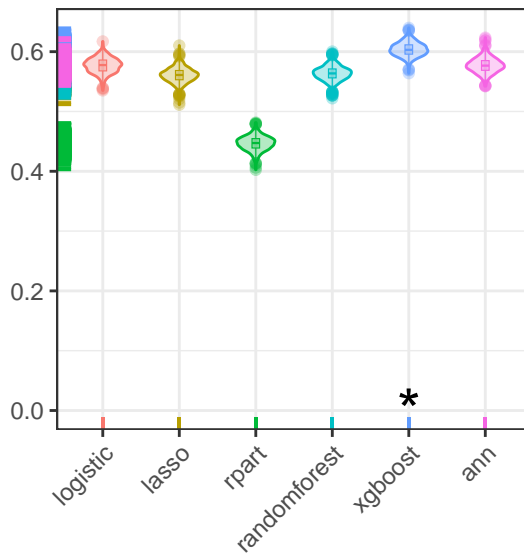

f1Score\_MIMIC-III

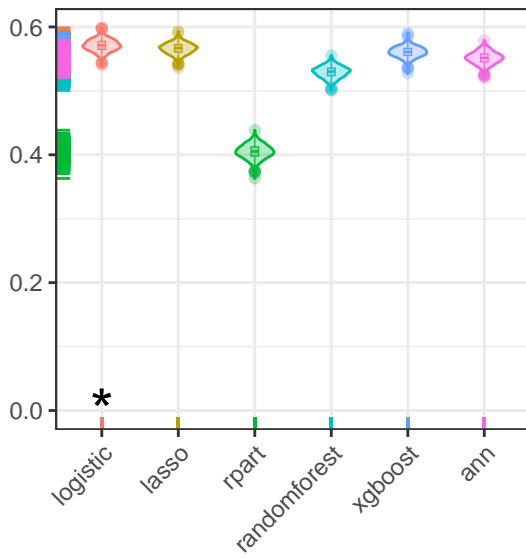

youden\_MIMIC-III

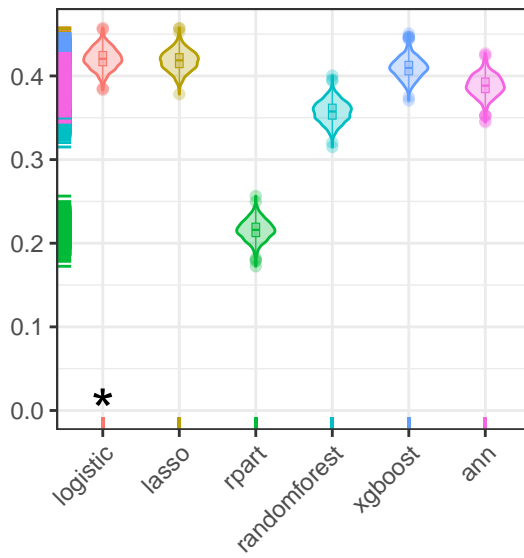

Supplement: Multimedia component 6 [file mmc6.pdf]

Brier\_MIMIC-III

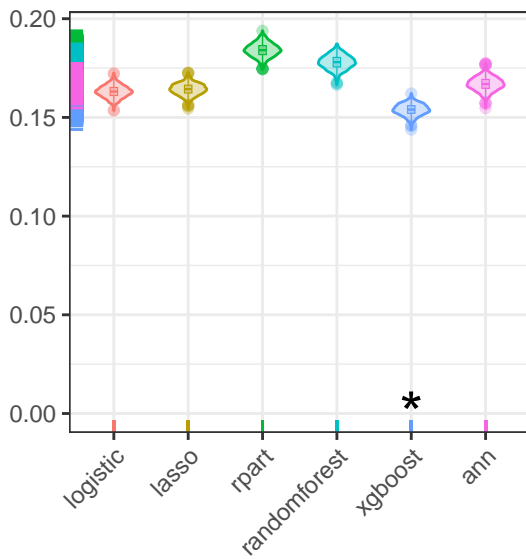

kappa\_MIMIC-III

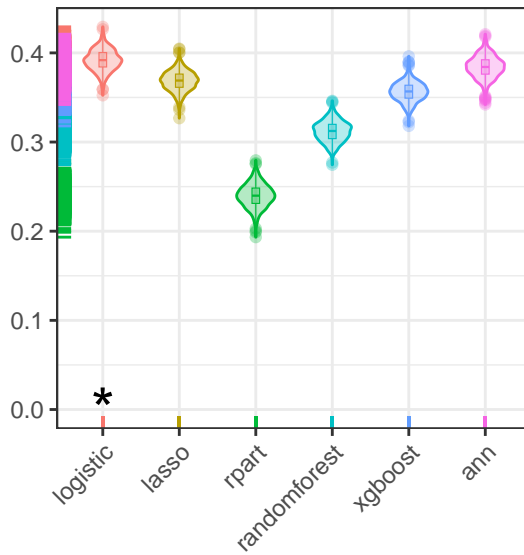

IDI\_MIMIC-III

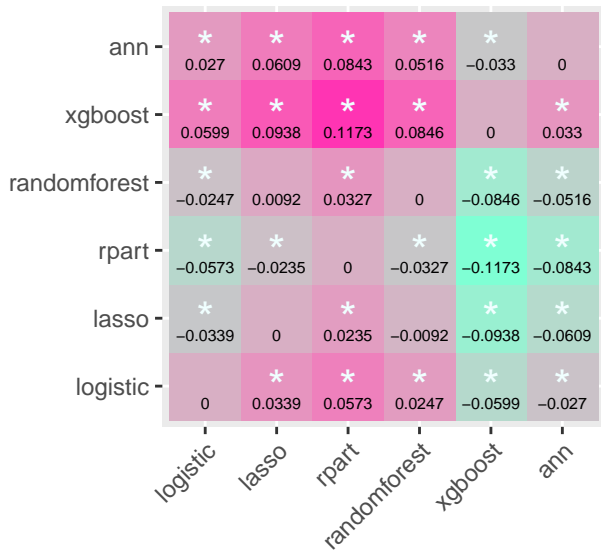

roc.test\_MIMIC-III

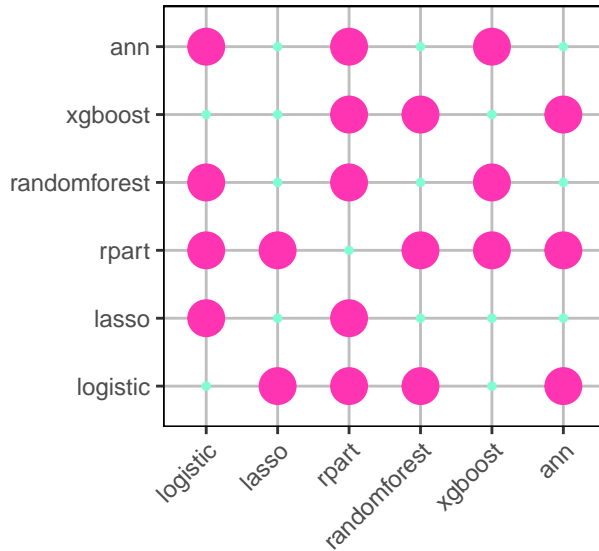

Supplement: Multimedia component 7 [file mmc7.pdf]

AUROC\_BFH

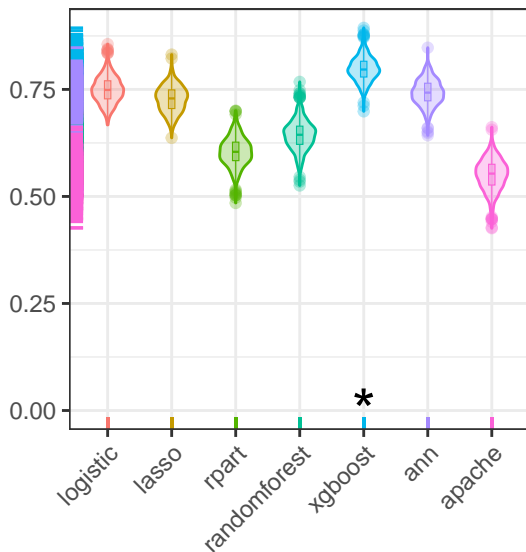

AUPRC\_BFH

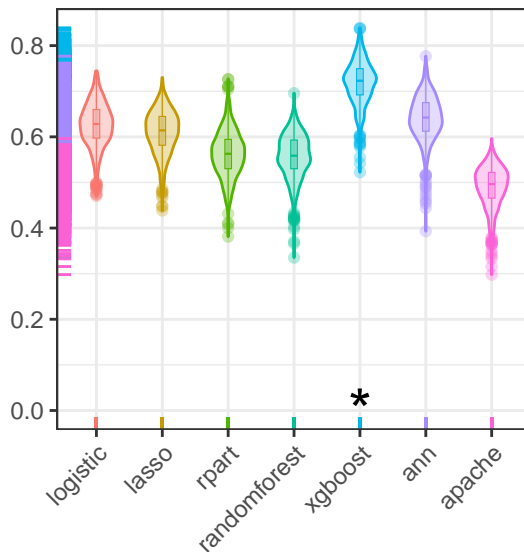

f1Score\_BFH

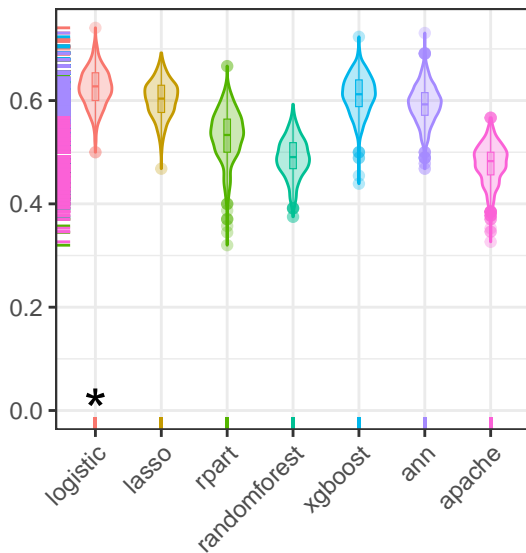

youden\_BFH

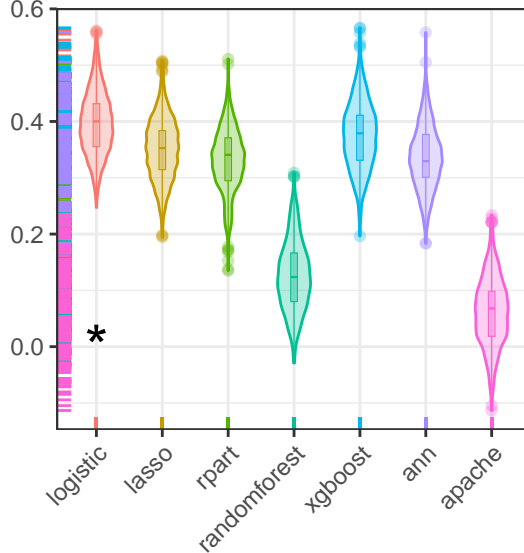

Supplement: Multimedia component 8 [file mmc8.pdf]

### Brier\_BFH

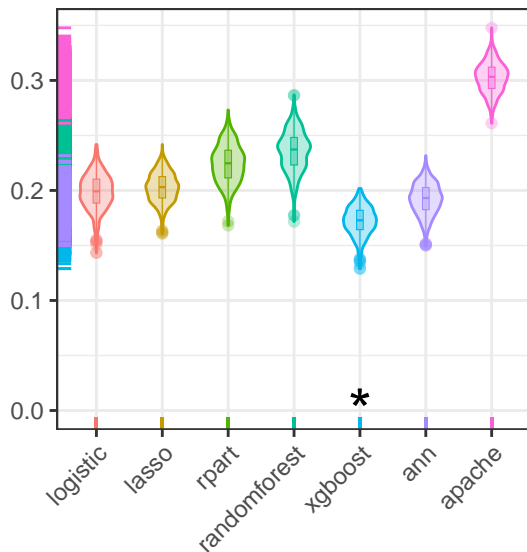

### kappa\_BFH

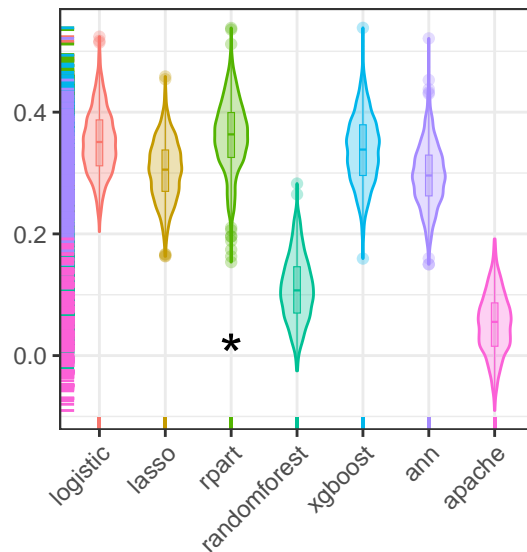

### IDI\_BFH

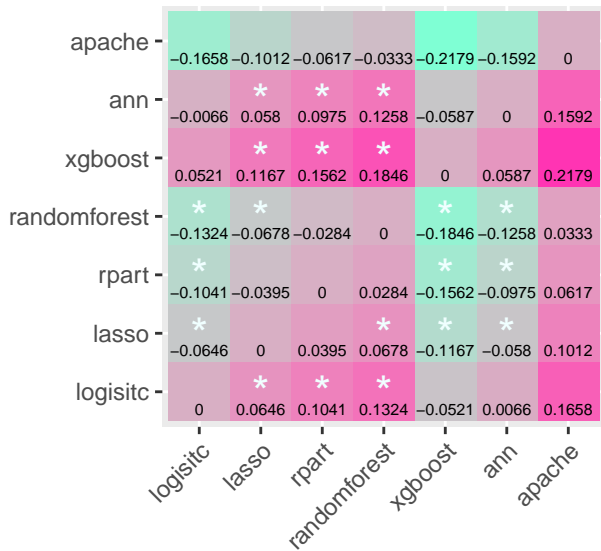

### roc.test\_BFH

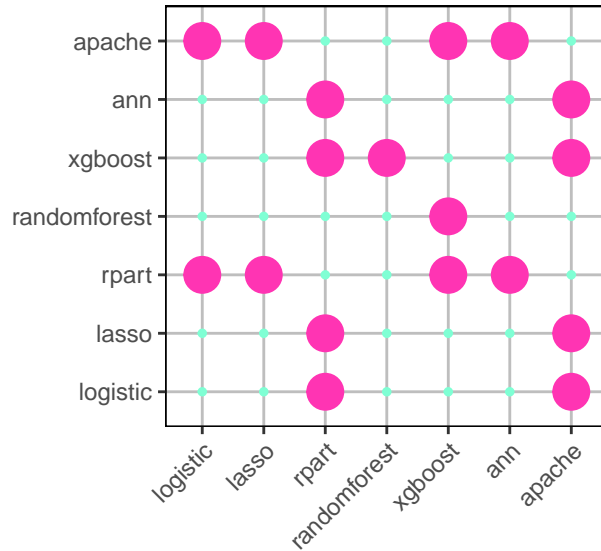

Supplement: Multimedia component 9 [file mmc9.pdf]
