# Supplementary material for: Development and validation of a machine-learning model for predicting the risk of death in sepsis patients with acute kidney injury
Source: Heliyon. 2024 Apr 20;10(9):e29985. doi: 10.1016/j.heliyon.2024.e29985 (PMC11064448; doi:10.1016/j.heliyon.2024.e29985)
Supplement: Multimedia component 10 [file mmc10.docx]

**Supplementary Figure 1.** The differences between the six models in terms of the area under the receiver operating characteristic (AUROC) curve, area under the precision-recall curve (AUPRC), f1score, and youden_index in the training set.

**Supplementary Figure 2.** The differences between the six models in terms of the Brier score and kappa coefficient. The integrated discrimination improvement (IDI) and receiver operating characteristic (ROC) test between every two models. In the IDI diagram, the x-axis represents the original model while the y-axis represents the new model, and the IDI value between them (* denotes p<0.05, a positive value indicates model improvement, while a negative value indicates the opposite). In the ROC diagram, the large pink dots denotes p<0.05, which is defined as having a difference.

**Supplementary Figure 3.** The differences between the six models in terms of the area under the receiver operating characteristic (AUROC) curve, area under the precision-recall curve (AUPRC), f1score, and youden_index in the internal validation.

**Supplementary Figure 4.** The differences between the six models in terms of the Brier score and kappa coefficient in the internal validation. The integrated discrimination improvement (IDI) and receiver operating characteristic (ROC) test between every two models.

**Supplementary Figure 5.** The differences between the six models in terms of receiver operating characteristic (ROC) curve analysis, precision-recall curve (PRC) analysis, calibration, and decision curve analysis (DCA) using the validation set MIMIC-III.

**Supplementary Figure 6.** The differences between six models in terms of the area under the receiver operating characteristic (AUROC) curve, area under the precision-recall curve (AUPRC), f1score, and youden_index using the validation set MIMIC-III.

**Supplementary Figure 7.** The differences between the six models in terms of the Brier score and kappa coefficient using the validation set MIMIC-III. The integrated discrimination improvement (IDI) and receiver operating characteristic (ROC) test between every two models.

**Supplementary Figure 8:** The differences between six models in terms of AUROC, AUPRC, f1score, and youden_index in the validation set BFH.

**Supplementary Figure 9.** The differences between the six models in terms of the Brier score and kappa coefficient using the validation set BFH. The integrated discrimination improvement (IDI) and receiver operating characteristic (ROC) test between every two models.
