# Supplementary material for: Development and validation of a machine-learning model for predicting the risk of death in sepsis patients with acute kidney injury
Source: Heliyon. 2024 Apr 20;10(9):e29985. doi: 10.1016/j.heliyon.2024.e29985 (PMC11064448; doi:10.1016/j.heliyon.2024.e29985)
Supplement: Multimedia component 11 [file mmc11.pdf]

Supplementary Table 1.Characteristics of the population

	Train	Test	MIMIC-III	BFH	p	test	Missing
n	4001	1714	1757	72			
hospital_expire_flag = death (%)	886 (22.1)	389 (22.7)	469 (26.7)	25 (34.7)	<0.001		0
Septic shock = 1 (%)	507 (12.7)	215 (12.5)	480 (27.3)	55 (76.4)	<0.001		0
Unspecified essential hypertension = 1 (%)	1129 (28.2)	457 (26.7)	667 (38.0)	39 (54.2)	<0.001		0
Diabetes mellitus without mention of complication, type II = 1 (%)	537 (13.4)	231 (13.5)	363 (20.7)	32 (44.4)	<0.001		0
Congestive heart failure, unspecified = 1 (%)	616 (15.4)	293 (17.1)	547 (31.1)	5 (6.9)	<0.001		0
Acute posthemorrhagic anemia = 1 (%)	603 (15.1)	241 (14.1)	0 (0.0)	4 (5.6)	<0.001		0
Acute respiratory failure with hypoxia = 1 (%)	521 (13.0)	237 (13.8)	0 (0.0)	40 (55.6)	<0.001		0
Acute respiratory failure = 1 (%)	904 (22.6)	379 (22.1)	863 (49.1)	40 (55.6)	<0.001		0
heart_rhythm_af = 1 (%)	384 (9.6)	179 (10.4)	201 (11.4)	9 (12.5)	0.17		0
heart_rhythm_A_Paced = 1 (%)	241 (6.0)	87 (5.1)	19 (1.1)	0 (0.0)	<0.001		0
heart_rhythm_AV_Paced = 1 (%)	121 (3.0)	57 (3.3)	21 (1.2)	0 (0.0)	<0.001		0
heart_rhythm_LBBB = 1 (%)	8 (0.2)	6 (0.4)	4 (0.2)	0 (0.0)	0.723		0
heart_rhythm_ST = 1 (%)	1021 (25.5)	438 (25.6)	528 (30.1)	0 (0.0)	<0.001		0
heart_rhythm_VT = 1 (%)	3 (0.1)	1 (0.1)	2 (0.1)	0 (0.0)	0.935		0
ventilation_statusInvasiveVent = 1 (%)	2827 (70.7)	1205 (70.3)	1349 (76.8)	30 (41.7)	<0.001		0
anchor_age (median [IQR])	65.00 [55.00, 75.00]	65.00 [55.00, 75.00]	65.92 [54.08, 76.68]	64.00 [48.00, 77.00]	0.176	nonnorm	0
lactate_max_gas (median [IQR])	2.70 [1.70, 4.60]	2.60 [1.70, 4.50]	2.40 [1.50, 4.40]	2.25 [1.37, 4.10]	<0.001	nonnorm	0
po2_max_gas (median [IQR])	241.00 [142.00, 373.00]	228.00 [139.25, 364.00]	216.00 [139.00, 330.00]	113.00 [81.00, 141.25]	<0.001	nonnorm	0
pco2_min_gas (median [IQR])	34.00 [30.00, 38.00]	34.00 [30.00, 39.00]	34.00 [29.00, 39.00]	34.65 [28.00, 45.25]	0.192	nonnorm	0
pco2_max_gas (median [IQR])	47.00 [41.00, 54.00]	47.00 [41.00, 55.00]	34.00 [29.00, 39.00]	34.65 [28.00, 45.25]	<0.001	nonnorm	0
baseexcess_min_gas (median [IQR])	-4.00 [-9.00, -1.00]	-4.00 [-9.00, -1.00]	-5.00 [-10.00, -1.00]	-3.70 [-7.53, 0.68]	0.006	nonnorm	0
baseexcess_max_gas (median [IQR])	0.00 [-2.00, 2.00]	0.00 [-2.00, 3.00]	0.00 [-3.00, 3.00]	-3.70 [-7.53, 0.68]	<0.001	nonnorm	0
calcium_min_gas (median [IQR])	1.06 [0.99, 1.12]	1.06 [0.99, 1.11]	1.05 [0.98, 1.12]	1.04 [0.97, 1.11]	0.192	nonnorm	0
calcium_max_gas (median [IQR])	1.16 [1.10, 1.23]	1.15 [1.09, 1.22]	1.15 [1.09, 1.21]	1.04 [0.97, 1.11]	<0.001	nonnorm	0
gcs_min (median [IQR])	13.00 [8.00, 14.00]	13.00 [8.00, 15.00]	15.00 [12.00, 15.00]	15.00 [9.00, 15.00]	<0.001	nonnorm	0
heart_rate_max (median [IQR])	107.00 [93.00, 124.00]	106.00 [93.00, 122.00]	110.00 [95.00, 127.00]	118.50 [107.50, 138.25]	<0.001	nonnorm	0
sbp_min (median [IQR])	84.00 [75.00, 93.00]	85.00 [76.00, 94.00]	84.00 [74.00, 94.00]	99.50 [90.75, 108.25]	<0.001	nonnorm	0
resp_rate_min (median [IQR])	12.00 [9.50, 15.00]	12.00 [9.00, 15.00]	12.00 [10.00, 15.00]	19.00 [16.00, 23.00]	<0.001	nonnorm	0
resp_rate_max (median [IQR])	28.00 [24.00, 32.00]	28.00 [24.00, 32.00]	28.00 [24.00, 33.00]	19.00 [16.00, 23.00]	<0.001	nonnorm	0
temperature_max (median [IQR])	37.44 [37.06, 38.06]	37.50 [37.06, 38.11]	37.56 [37.00, 38.22]	38.00 [37.18, 38.50]	0.005	nonnorm	0
spo2_min (median [IQR])	93.00 [90.00, 95.00]	93.00 [90.00, 95.00]	92.00 [89.00, 95.00]	96.50 [94.00, 100.00]	<0.001	nonnorm	0
glucose_min.x (median [IQR])	101.00 [85.00, 125.00]	102.00 [84.25, 128.00]	107.00 [88.00, 131.00]	165.78 [118.17, 269.19]	<0.001	nonnorm	0
urineoutput (median [IQR])	1175.00 [700.00, 1808.00]	1180.00 [677.75, 1810.00]	1210.00 [657.00, 1965.00]	1832.50 [570.25, 2643.75]	0.009	nonnorm	0
platelets_min (median [IQR])	149.00 [99.00, 212.00]	151.50 [101.00, 213.00]	164.00 [96.00, 236.00]	96.50 [52.00, 161.75]	<0.001	nonnorm	0
aniongap_max (median [IQR])	17.00 [14.00, 21.00]	17.00 [14.00, 20.00]	17.00 [14.00, 20.00]	17.85 [15.25, 21.30]	0.071	nonnorm	0
bun_max (median [IQR])	25.00 [17.00, 39.00]	25.50 [18.00, 41.00]	32.00 [20.00, 53.00]	16.64 [11.69, 31.40]	<0.001	nonnorm	0
calcium_max (median [IQR])	8.40 [8.00, 9.00]	8.45 [8.00, 8.90]	8.50 [8.00, 9.10]	7.72 [7.20, 8.28]	<0.001	nonnorm	0
chloride_max (median [IQR])	107.00 [103.00, 111.00]	107.00 [103.00, 111.00]	108.00 [104.00, 112.00]	104.50 [100.00, 110.25]	<0.001	nonnorm	0
creatinine_max (median [IQR])	1.30 [0.90, 2.20]	1.30 [0.90, 2.20]	1.50 [1.00, 2.70]	2.57 [1.50, 4.08]	<0.001	nonnorm	0
pt_min (median [IQR])	13.50 [12.20, 15.70]	13.50 [12.30, 15.90]	14.20 [12.90, 16.40]	14.70 [13.57, 17.20]	<0.001	nonnorm	0
pt_max (median [IQR])	15.60 [13.60, 20.00]	15.50 [13.60, 19.60]	16.20 [13.90, 21.30]	14.70 [13.57, 17.20]	<0.001	nonnorm	0
ptt_min (median [IQR])	29.00 [25.90, 33.50]	29.10 [26.10, 33.90]	29.50 [25.90, 34.90]	37.60 [29.77, 46.18]	<0.001	nonnorm	0
ptt_max (median [IQR])	35.30 [29.30, 51.30]	35.40 [29.50, 52.05]	37.50 [29.80, 58.50]	37.60 [29.77, 46.18]	0.001	nonnorm	0

Chisq.test() is used for categorical variables (with continuity correction) and oneway.test() is used for continous variables (with equal variance assumption, i.e., regular ANOVA).And kruskal.test() is used for the nonnormal continous variables and fisher.test() is used for categorical variables specified in the exact argument.
